# Pulmonary function testing in preoperative high-risk patients

**DOI:** 10.1186/s13741-024-00368-w

**Published:** 2024-03-05

**Authors:** Christine Eimer, Natalia Urbaniak, Astrid Dempfle, Tobias Becher, Dirk Schädler, Norbert Weiler, Inéz Frerichs

**Affiliations:** 1 University Medical Center Schleswig-Holstein, Anesthesiology and Intensive Care Medicine, Arnold-Heller Str. 3, 24105 Kiel, Germany; 2 Christian-Albrechts University, Institute of Medical Informatics and Statistics, Brunswikerstr. 10, 24105 Kiel, Germany

**Keywords:** Spirometry, Airflow obstruction, D_LCO_, Preoperative screening, Patient safety, Intensive care medicine, Postoperative pulmonary complications, Postoperative respiratory failure, Targeted screening program, Pulmonary gas exchange

## Abstract

**Background:**

Postoperative respiratory failure is the most frequent complication in postsurgical patients. The purpose of this study is to assess whether pulmonary function testing in high-risk patients during preoperative assessment detects previously unknown respiratory impairments which may influence patient outcomes.

**Methods:**

A targeted patient screening by spirometry and the measurement of the diffusing capacity of the lung for carbon monoxide (D_LCO_) was implemented in the anesthesia department of a tertiary university hospital. Patients of all surgical disciplines who were at least 75 years old or exhibited reduced exercise tolerance with the metabolic equivalent of task less than four (MET < 4) were examined. Clinical characteristics, history of lung diseases, and smoking status were also recorded. The statistical analysis entailed *t*-tests, one-way ANOVA, and multiple linear regression with backward elimination for group comparisons.

**Results:**

Among 256 included patients, 230 fulfilled the test quality criteria. Eighty-one (35.2%) patients presented obstructive ventilatory disorders, out of which 65 were previously unknown. 38 of the newly diagnosed obstructive disorders were mild, 18 moderate, and 9 severe. One hundred forty-five D_LCO_ measurements revealed 40 (27.6%) previously unknown gas exchange impairments; 21 were mild, 17 moderate, and 2 severe. The pulmonary function parameters of forced vital capacity (FVC), forced expiratory volume in 1 s (FEV_1_), and D_LCO_ were significantly lower than the international reference values of a healthy population. Patients with a lower ASA class and no history of smoking exhibited higher FVC, FEV_1_, and D_LCO_ values. Reduced exercise tolerance with MET < 4 was strongly associated with lower spirometry values.

**Conclusions:**

Our screening program detected a relevant number of patients with previously unknown obstructive ventilatory disorders and impaired pulmonary gas exchange. This newly discovered sickness is associated with low metabolic equivalents and may influence perioperative outcomes. Whether optimized management of patients with previously unknown impaired lung function leads to a better outcome should be evaluated in multicenter studies.

**Trial registration:**

German Registry of Clinical Studies (DRKS00029337), registered on: June 22nd, 2022.

## Background

The most common anesthesia-related complications involve the cardiovascular and the respiratory systems, making it particularly important to assess patient risk specifically in relation to these organ systems. Postoperative respiratory failure (PRF) frequently occurs in postsurgical patients (Canet and Gallart 2014; Mills 2018). PRF is associated with increased hospital length of stay, increased healthcare costs, higher morbidity, and mortality (David et al. 2017; Zettervall et al. 2017; Bostock et al. 2018). The incidence of PRF varies from 0.2 to 4.2% (Canet and Gallart 2014; Canet et al. 2015), with even higher rates in cardiac surgery of 2.6–8.0% (Thanavaro et al. 2020; Liu et al. 2018; Shoji et al. 2017; García-Delgado et al. 2012). Recognition and prevention of postoperative pulmonary complications (PPC) is an important research priority in intensive care medicine (Gillies et al. 2017).

The pathogenesis of respiratory failure depends on different aspects, such as patient status, type of anesthesia, and surgical procedures (Canet and Gallart 2014; Mills 2018). Improving patient safety has been a focus of clinical care ever since, therefore the goal of risk evaluation is to detect previously unknown or inadequately treated diseases that could be of significance for the anesthesia or surgical procedure or postoperative care. In recent years, numerous studies have been conducted with the aim of identifying factors promoting PPC. Among those ASA class > 3, emergency or high-risk surgery, type of surgery, functional status, heart failure or chronic pulmonary disease, obesity, and older age were identified as risk factors for pulmonary complications (Attaallah et al. 2019; Brueckmann et al. 2013; Gupta et al. 2013).

The preoperative evaluation of the lungs and airways serves to diagnose previously unknown lung function impairments. Pulmonary function testing can be used to quantitatively assess lung function and to specify the different forms of lung diseases (Bernstein 2012). The purpose of this study was to assess whether pulmonary function testing in preoperative high-risk patients identified patients with previously unknown relevant pulmonary function deterioration.

## Methods

**Fig. 1:**
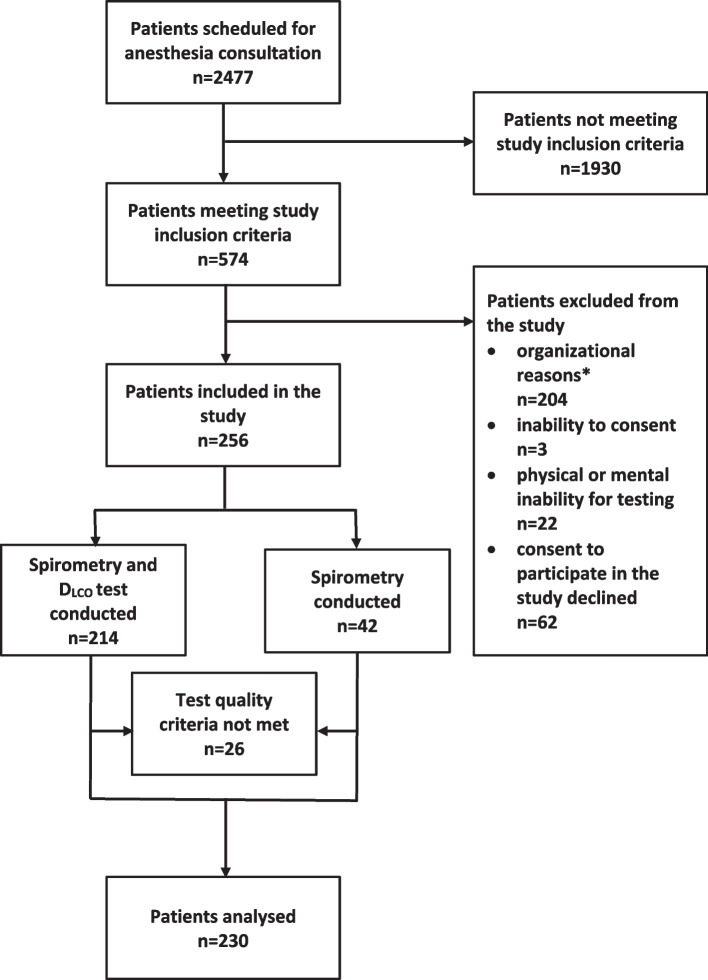
Study inclusion flow chart. Legend: *Prior lung function testing less than 3 months ago *n* = 25, canceled operative procedure *n* = 27, patient consulting the anesthesia out-patient clinic for the second time within the study period *n* = 23, anesthesia consultation not held in out-patient clinic *n* = 13, time shortage *n* = 86, missing devices for the spirometer *n* = 30

This monocentric, prospective observational study was conducted in the Department of Anesthesiology and Intensive Care Medicine at the University Medical Centre Schleswig–Holstein, Campus Kiel after prior approval by the Ethics Committee of the Medical Faculty of the Kiel University (D580/20). The study was registered in the German Clinical Trials Register (DRKS) (DRKS00029337). Patients were informed verbally, participation was voluntary, and written consent was not required. Patients of all surgical disciplines with an age ≥ 75 years or an exercise capacity below four metabolic equivalents of task (MET) were included in the study. Exclusion criteria were a lack of consent, age < 18 years or inability to complete the study due to cognitive or physical disabilities, or available prior spirometry performed less than 3 months ago (Fig. 1). Spirometry parameters (FVC: Forced vital capacity, FEV_1_: Forced expiratory volume in one second) and diffusing capacity of the lung for carbon monoxide (D_LCO_) were used to examine the lung function (EasyOne Pro, ndd, Zurich, Switzerland). Age, sex, body height and weight, smoking status, ASA classification, exercise capacity in METs, and known pulmonary diseases were recorded (Table 1). Spirometric parameters were used to diagnose obstructive ventilatory disorders and pulmonary gas exchange impairments and were categorized into severity levels according to the European Respiratory Society/American Thoracic Society (ERS/ATS) standards (Stanojevic et al. 2022). The severity grade of ventilatory obstruction was categorized as grades I, II, and III with > 60%, 40–60%, and < 40% of the FEV_1_% predicted. The severity of decrease in diffusing capacity for carbon monoxide was categorized as grade I, II, and III with > 60% and < 80%, 40–60%, and < 40% of the DLCO% predicted. Furthermore, the Global Lung Function Initiative (GLI) 2012 reference values were applied and *Z*-scores for each parameter calculated (Quanjer et al. 2012). FVC or FEV_1_ *Z*-score <  − 1.645 (corresponding to the 5th population percentile) was defined as impaired lung function and a D_LCO_ *Z*-score <  − 1645 was also defined as impaired diffusing capacity. Patient-related data was anonymized after data collection to ensure data protection.

**Table 1:** Demographic and clinical characteristics

	**Total number n or mean ± SD**
**Patients**	230
**Female**	107 (46.5%)
**Male**	123 (53.5%)
**Age, y**ears	75 ± 11 (min. 24, max. 90)
**Height, cm**	170 ± 8
**Weight, kg**	78 ± 19
**BMI, kg/m**^**2**^	27 ± 6
**Normal weight: BMI 18.5**–**24.9 (kg/m**^**2**^**)**	88 (38.3%)
**Overweight: BMI 25**–**29.9 (kg/m**^**2**^**)**	86 (37.4%)
**Obesity: ≥ 30 (kg/m**^**2**^**)**	50 (21.7%)
**Underweight: BMI < 18.5 (kg/m**^**2**^**)**	6 (2.6%)
**MET < 4**	101 (43.9%)
**MET ≥ 4**	129 (56.1%)
**ASA 1**	6 (2.6%)
**ASA 2**	91 (39.6%)
**ASA 3**	125 (54.3%)
**ASA 4**	8 (3.5%)
**ASA 5**	0
**Packyears**	16 ± 22
**Current smokers**	44 (19.1%)
**Former smokers**	99 (43.0%)
**Never smokers**	87 (37.8%)
**Known lung disease**	28 (12.2%)

### Spirometry

The spirometry measurements were conducted with a nose clip, the patient was asked to breathe in through the open mouth as deeply as possible and to breathe out as forcefully as possible through the mouthpiece of the spirometer. This procedure was conducted at least twice. Only lung function tests with a quality grade A-D were included in the study. For the D_LCO_ measurement patients were asked to breathe normally, to exhale completely, and then to take a deep breath. During the deep inspiration, a test gas (9.34% helium, 0–269% carbon monoxide, 19.11% oxygen and 71.281% nitrogen) was added. After holding the breath for approximately 10 s the patient breathed out and the exhaled gas was analyzed. The examination was repeated once if it did not meet the quality criteria (Stanojevic et al. 2022).

### Statistical analysis

To investigate whether lung function in a high-risk clinical sample differs from the general healthy population, one-sample *t*-tests on *Z*-scores for FVC, FEV_1_, and D_LCO_ were performed. FVC, FEV_1_, and D_LCO_ Z-scores were compared between patients with MET < 4 vs MET ≥ 4 using two-sample t-tests. To analyze the association between ASA classification and lung function, ASA classes were combined (ASA 1–2; ASA 3–4) and compared regarding FVC, FEV_1_, and D_LCO_ Z-scores using two-sample *t*-tests. To investigate the influence of smoking status on lung function, subjects were categorized as “never smokers” (NS) “former smokers” (FS), and “current smokers” (CS) and one-way ANOVAs on FVC, FEV_1_, and D_LCO_ *Z*-scores were computed. The association between body mass index (BMI) and FVC, FEV_1_, and D_LCO_ *Z*-scores were analyzed using linear regression considering all patients with BMI between 18.5 kg/m^2^ and 45 kg/m^2^. To investigate which of the considered clinical characteristics had independent effects on lung function, multiple linear regression models with backward elimination were performed with the respective *Z*-scores as dependent variable, and metabolic equivalents (2 categories), BMI, ASA class, smoking (quantified in pack-years) and known lung disease as explanatory variables. Statistical analysis was performed using SPSS Statistics 29 Software, IBM (Armonk, NY, USA), and figures were created with GraphPad Software, Prism Version 9.3.1 (San Diego, USA).

## Results

The detailed study inclusion and exclusion flowchart is presented in Fig. 1. Five hundred forty-seven patients met the study inclusion criteria. Pulmonary function testing was performed on 256 of the eligible study participants, among which 230 met pulmonary test quality criteria. Clinical characteristics are shown in Table 1. Two hundred fourteen patients underwent both, the spirometry examination and the D_LCO_ testing (Fig. 1)_._ Forty-two patients completed only the forced full expiration maneuver because of a temporary shortage of D_LCO_ filters.

**Fig. 2:**
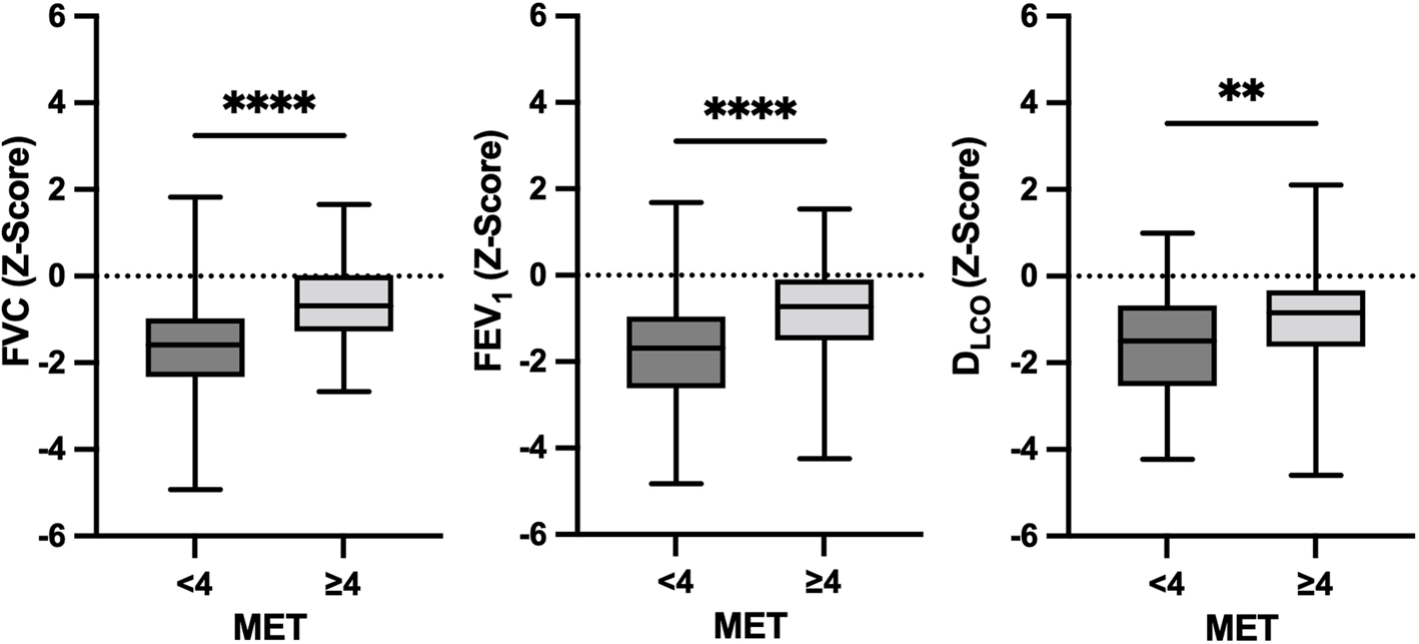
Boxplots of lung function parameters in relation to metabolic equivalent of task (MET)

**Fig. 3:**
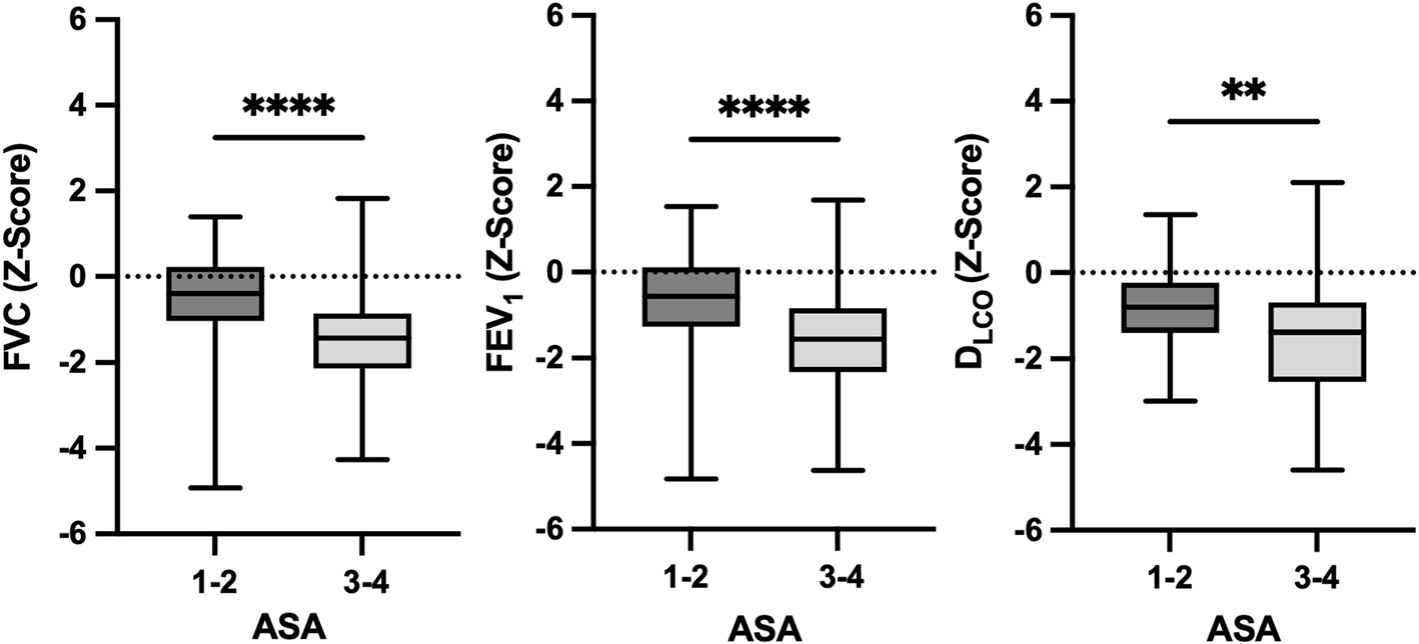
Boxplots of lung function parameters in relation to ASA classification

**Fig. 4:**
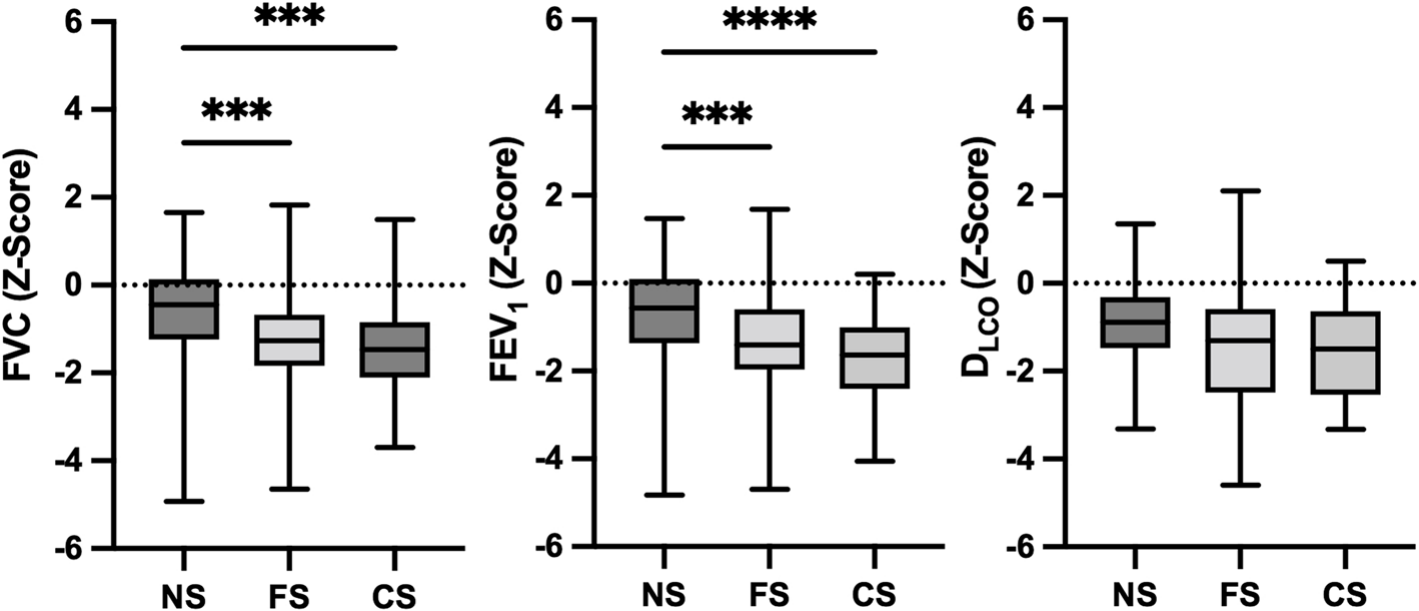
Boxplots of lung parameters examined in relation to smoking status

Among 230 patients whose pulmonary function tests met the quality criteria, 81 (35.2%) had obstructive ventilatory disorders. Sixty-five of these identified obstructive disorders were previously unknown, 38 of them were mild, 18 moderate, and 9 were severe. Out of 145 analyzed D_LCO_ measurements, 40 (27.6%) revealed previously unknown pulmonary gas exchange impairments with 21 being mild, 17 moderate, and 2 severe. The selected *Z*-scores were significantly different from population norms (with expected mean equal to zero) with mean FVC *Z*-score of 1.05 (SD 1.19), mean FEV_1_ Z-score of − 1.22 (SD 1.29), and mean D_LCO_ Z-score of − 1.24 (SD 1.21) (all *p* < 0.001, one sample *t* test). One hundred one of the patients had less than four metabolic equivalents and 129 had at least four metabolic equivalents. Two sample tests between the patient group of MET ≥ 4 and the group with MET < 4 showed significantly different results for the FVC (*p* < 0.001), FEV_1_ (*p* < 0.001), and D_LCO_ (*p* < 0.004) *Z*-scores. The MET < 4 group exhibited significantly lower *Z*-scores than the MET ≥ 4 group (Fig. 2, Table 2). Two-sample *t*-tests between the two ASA groups showed significantly different means of the two groups for all three *Z*-scores (FVC *p* < 0.001, FEV_1_ *p* < 0.001, D_LCO_ *p* < 0.002). Patients with a lower ASA classification had significantly better lung function (Fig. 3, Table 2). Twelve (15.2%) patients showed impaired lung function results, and 9 (18%) patients had pathological diffusing capacity despite MET ≥ 4 and ASA classification 1–2. Smoking imposed a significant effect on lung function parameters (FVC *p* < 0.001, FEV_1_ *p* < 0.001, D_LCO_ *p* = 0.019) and Z-scores were the highest in the “never smoking” group and the lowest in the group of current smokers (Fig. 4, Table 2).

**Table 2:** Pulmonary function parameters in the studied patients

	FVC (mean ± SD)	FEV_1_ (mean ± SD)	D_LCO_ (mean ± SD)
**MET < 4**	− 1.58 ± 1.28	− 1.82 ± 1.36	− 1.57 ± 1.28
**MET ≥ 4**	− 0.64 ± 0.92	− 0.75 ± 1.00	− 0.99 ± 1.10
**ASA 1–2**	− 0.52 ± 1.13	− 0.64 ± 1.15	− 0.88 ± 0.93
**ASA 3–4**	− 1.44 ± 1.07	− 1.65 ± 1.21	− 1.51 ± 1.33
**Never smokers**	− 0.62 ± 1.15	− 0.71 ± 1.2	0.92 ± 0.99
**Former smokers**	− 1.25 ± 1.13	− 1.44 ± 1.32	− 1.42 ± 1.38
**Current smokers**	− 1.45 ± 1.13	− 1.74 ± 1.02	− 1.49 ± 1.15

*Abbreviations*: *FVC* forced vital capacity, *FEV*_*1*_ forced expiratory volume in 1 s, *D*_*lco*_ diffusing capacity of the lung for carbon monoxide, *MET* metabolic equivalents of task, *BMI* body mass index

**Fig. 5:**
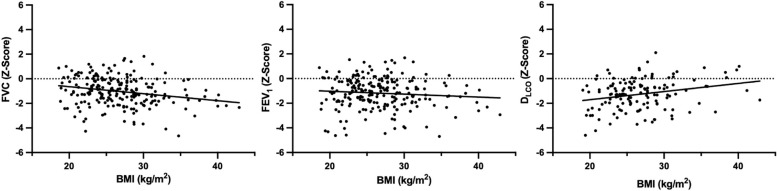
Scatter plots of the lung parameters examined in relation to body mass index (BMI). Legend: Boxplots (Figs. 2, 3, and 4) show the distribution of lung function parameters in relation to MET, ASA, and smoking status. Whiskers show the minimum and maximum of the values, the horizontal line in the boxes represents the median. Stars represent the level of significance, whereas **** is equivalent to *p* < 0.0001 and ** is equivalent to *p* < 0.01. Scatter plots (Fig. 5) show the relationship between lung function parameters and BMI with regression lines indicating the overall trend of data. Correlation: FVC: *r* =  − 0.2319, *p* = 0.001; FEV_1_: *r* =  − 0.0886, *p* = 0.189; D_LCO_: *r* = 0.2570, *p* = 0.002. Abbreviations: *FVC* forced vital capacity; *FEV*_*1*_ forced expiratory pressure in 1 s; *D*_*LCO*_ diffusing capacity of the lung for carbon monoxide; *MET* metabolic equivalents of task; *NS* never smoker; *FS* former smoker; *CS* current smoker; *BMI* body mass index

BMI showed a significant negative correlation with the FVC *Z*-score (*p* < 0.001), a slight however non-significant negative correlation with FEV_1_ Z-score (*p* = 0.189), and a significant positive correlation with the D_LCO_ *Z*-score (*p* = 0.002) (Fig. 5).

Multiple linear regression with backward elimination indicates reduced exercise tolerance with MET < 4 and smoking history (quantified as the number of packyears) as significant variables in statistical models for all three *Z*-scores. ASA classification, known lung disease, and BMI were not significant for at least one of the *Z*-scores.

## Discussion

The preoperative lung function screening of high-risk patients revealed a high number of previously unknown ventilatory obstructive disorders and pulmonary gas exchange impairments. Obstructive disorders were newly diagnosed in 28% of 230 analyzed study participants. An equal proportion of 28% out of the 145 patients who underwent the D_LCO_ examination showed an impaired gas exchange. Lower *Z*-scores in both spirometry parameters and D_LCO_ were significantly associated with reduced exercise tolerance with MET < 4. A relevant proportion of lung function measurements and DLCO measurements showed pathological results in patients with MET > 4 and ASA 1–2, who would not have been identified as at-risk patients based on MET and ASA classification alone. These results suggest that additional determination of lung function and DLCO may provide additional information in preoperative risk assessment.

The time required for pulmonary function testing (2–3 attempts) and the measurement of diffusion capacity (1–2 attempts) is approximately 15 min per patient. Regarding the daily feasibility of the examinations, the aim of this study was to define a specific patient cohort that would indeed benefit from a corresponding measurement.

PPC occur more frequently and results in greater costs than cardiovascular events (Brinson and Thornton 2018). Patients requiring unplanned postoperative ventilatory support have higher morbidity and mortality rates (Magor et al. 2022). Robitaille et al. implemented a targeted screening program to detect airflow obstruction within a presurgical screening and found previously unknown obstructive disorders in 26% of all study participants (Robitaille et al. 2015). Spirometry examinations performed as part of a lung cancer screening program also detected a high prevalence of airflow obstruction in individuals without prior diagnosis of COPD (Balata et al. 2020). Similar to the present study, these results suggest the need for intensified preoperative screenings so that newly discovered sicknesses can be recognized and treated preoperatively. However, studies have shown different results regarding the usefulness of lung function testing to predict PPC. A Nigerian study conducted a pre-operative pulmonary assessment and found an abnormally low percentage of FEV_1_ and FVC to be significantly associated with PPC (Ufoaroh et al. 2019). Another spirometry test before laparoscopy-assisted gastrectomy showed that preoperative spirometry is not reliable in predicting PPCs (Huh et al. 2013). Regarding operative procedures of the aorta, spirometry results had a predictive value regarding mortality (Henn et al. 2016).

This study analyzed the lung parameters of patients from a wide range of operative procedures ranging from simple urologic procedures to very demanding cardiac surgery. Due to this heterogeneity, the lung function of many different patient groups could be examined. The heterogeneity of operative procedures, however, complicated the comparison of risk factors. The study population had a similar risk assessment regarding the status as a “high-risk patient” due to age or exercise capacity of less than four METs. The risk of developing PPCs, however, is also influenced by the different operative procedures (Canet et al. 2015). Thus, it should be investigated in further studies which patient group undergoing which operative procedures could benefit the most from preoperative spirometry testing with consideration of the individual patient outcome.

The examined patient population had a significantly poorer lung function in comparison to the general population, which might be due to the specific risk selection of patients who need surgery and have less than four metabolic equivalents (Iden et al. 2019). Poor lung function may be clinically expected in patients with reduced exercise tolerance. However, low MET are not always the consequence of merely poor lung function. Lower *Z*-scores in both spirometry parameters and D_LCO_ were significantly associated with reduced exercise tolerance (MET < 4). In multiple linear regression, MET < 4 proved to be an independent risk factor for lower spirometry parameters regardless of ASA classification, BMI, history of smoking, and known pulmonary disease. Recent studies suggest a positive relationship between physical fitness and lung function (Bédard et al. 2020; Hancox and Rasmussen 2018; Farkhooy et al. 2018; Fuertes et al. 2018; Luzak et al. 2017) which seems to exist in both adults and children (Bédard et al. 2020; Hancox and Rasmussen 2018). Additionally, patients with lower MET scores seem to have more comorbidities (Zientara et al. 2021). The D_LCO_ testing describes the ability of the lung to exchange gases and can be reduced in several diseases of the lung (Weibel 2017). A low D_LCO_ is associated with pulmonary hypertension since it can be the first sign of the respective disease in patients with parenchymal lung disease (Zou et al. 2020). A meta-analysis suggests that D_LCO_ might be an important measurement for COPD patients in terms of severity, exacerbation risk, mortality, emphysema domination, and presence of pulmonary hypertension (Ni et al. 2021). Our study results presented 19 moderate to severe D_LCO_ reductions in allegedly lung-healthy patients. Therefore, 13% of our patients had a lung pathology possibly relevant to both the operative procedure itself and the postoperative outcome. The statistical analysis revealed a low D_LCO_ to be associated with a higher ASA classification and hence more comorbidities. The ASA classifications proved to be significantly associated with lung function parameters in our study. ASA 1–2 achieved higher values than ASA group 3–4. As the ASA classification system classifies patients according to their comorbidities, it seems plausible that a higher ASA classification is associated with a worse lung function (Böhmer et al. 2021; Horvath et al. 2021).

The influence of obesity on lung function parameters shows inconsistent results. There are studies demonstrating reduced FVC, FEV_1_, and D_LCO_ values in obese patients but there are also studies that do not find a significant association which, however, might be due to small sample sizes (Davidson et al. 2014; Mehari et al. 2015; Pekkarinen et al. 2012). The decrease in FVC Z-score with higher BMI might be explained by a decrease in chest wall compliance due to higher intra-abdominal pressure (Bein 2018). The increase in DLCO with BMI in our study is surprising and requires careful interpretation. Lung-healthy current and former smokers show increased regional ventilation heterogeneity during forced expiration as measured by electrical impedance tomography (Vogt et al. 2019). Our study results support those findings with significant differences in lung function parameters FVC, FEV_1_, and D_LCO_ between current smokers, former smokers, and never-smokers. Never-smokers had the best results regarding lung function and current smokers had the worst.

## Limitations

Regarding the D_LCO_ measurement, we did not adjust for elevated carbon monoxide back-pressure in the blood which can be expected to occur in smoking patients. This may have led to a slight underestimation of D_LCO_ in these patients. Furthermore, we performed only one or two acceptable spirometry maneuvers and not three as suggested by the guidelines, which was due to the tight time scheduling of the patient appointments. In order to adequately address this limitation, only lung function tests with a quality grade of A-D were included. To determine exercise tolerance even more precisely, the 6-min walk test appears to be an adequate method for assessing exercise tolerance and it may be included in an extended pulmonary function and exercise endurance examination.

Due to the study design, the occurrence of postoperative pulmonary complications could not be evaluated. Firstly, because pathological findings were communicated to the anesthesiologist in charge of the patient and secondly, because the data were anonymized after data collection. Intraoperative ventilation parameters were not recorded in the present study. With regard to postoperative pulmonary complications, this should be considered in further studies.

Whether the treatment of patients with previously unknown impaired lung function leads to an improved outcome should be evaluated in adequately powered studies.

## Conclusion

A targeted lung function screening program detected a relevant number of previously unknown obstructive ventilatory disorders and gas exchange impairments in high-risk preoperative patients. These newly discovered sicknesses are associated with low metabolic equivalents. A relevant proportion of lung function measurements and DLCO measurements showed pathological results in patients with MET > 4 and ASA 1–2, who would not have been identified as at-risk patients based on MET and ASA classification alone. These results suggest that the determination of lung function and DLCO may provide additional information in preoperative risk assessment. Whether the treatment of patients with previously unknown impaired lung function leads to an improved outcome should be evaluated in adequately powered studies.

## Data Availability

The datasets used and/or analyzed during the current study are available from the corresponding author on reasonable request.

## Abbreviations

ASA: American Society of Anesthesiologists
ATS: American Thoracic Society
BMI: Body Mass Index
CO: Carbon monoxide
COPD: Chronic obstructive pulmonary disease
D_LCO_: Diffusion capacity of the lung for carbon monoxide
DRKS: German Clinical Trials Register
EPCO: European Perioperative Clinical Outcome
ERS: European Respiratory Society
FEV_1_: Forced expiratory volume in one second
FVC: Forced vital capacity
ICU: Intensive care unit
MET: Metabolic equivalent of task
PPC: Postoperative pulmonary complications
